# Flourishing with Shared Vitality: Education based on Aesthetic Experience, with Performance for Meaning

**DOI:** 10.1007/s11217-021-09767-8

**Published:** 2021-03-28

**Authors:** Christine Doddington

**Affiliations:** Homerton College, University of Cambridge, Pump Lodge, 406 Cherry Hinton Road, Cambridge, CB1 8BA UK

**Keywords:** Aesthetic, Flourishing, Embodied, Meaning, Performance, Experience

## Abstract

In this paper, I set an aspect of what it is to live a flourishing life against the backdrop of neo liberal trends that continue to influence educational policy across the globe. The view I set out is in sharp contrast to any narrow assumption that education’s main task is the measurement of high performing individuals who will thus contribute to an economically viable society. Instead, I explore and argue for a conception of what constitutes a flourishing life that is embedded in a more pragmatist analysis of what education may be. The argument begins with developing the centrality of embodiment to aesthetic sensibility and goes on to suggest how collective understanding of this within a community could constitute a sense of performance and thus contribute to the educational aim of developing a full and flourishing life. The argument is that this way of seeing life could have implications for educational practice, the role of the teacher and could help to reconfigure how education is experienced by the young.

## Introduction

The dominance of a neo-liberal stress on performativity in education is currently powerful and persistent and there are many indications that this general orientation will continue to influence policy. In this paper, I wish to explore and argue for something that stands somewhat against this view. In short, I argue for a humane, embodied and sense-enhanced life that strives for meaning, attachments and affiliations, with the suggestion that this way of seeing life could have profound implications for educational practice. The necessity to reconfigure education in these terms will initially take us briefly to look at current social trends and inevitably draw us back to ideas concerning flourishing lives and communities, and views on the nature of education.

The title of this special edition is especially timely. Following the Covid 19 pandemic and indeed even before that crisis, sense based, experiential aspects of living have been regularly advocated as therapeutic experiences for anxious and stressed ways of living and are seen as providing some kind of answer to how we might all lead more flourishing lives. Different dimensions of mindfulness, the value of contact with, and experience of nature, outdoor activities, and exercise are featured throughout the media and self-help publishing, as solutions to how we might increase physical, mental health and wellbeing. In schools, there have been strong arguments for considering the importance of ‘wellbeing’ for pupils because this is “inseparable from their overall capacity to learn and achieve, and to become confident, self-assured and active citizens” (Thorburn 2018). These various calls for what might be seen as different forms of flourishing can be set within notions of what it is to educate, and are at times aspirations for the individual, at others, for the social good. Therapeutic experiences are frequently promoted as an antidote either to the age-old emphasis on exclusively rational or calculative aspiration or are seen as a more general personal antidote to high paced and stressful lives. There seems to be a general emphasis suggesting that greater social and personal consciousness combined with fundamental shifts in attitudes and activity can enhance our humanity and release our full potential. And so, a timeless division between ‘Sense and Sensibility’ (Austen 1811) echoes a dichotomy deeply entrenched in our perception of how we might pursue a good life. Austen’s novel warns of making too simplistic a contrast of rational against emotional behaviour and while the opposition of rationality to emotion has long been challenged, a clearer understanding of what underlies the current wide-ranging claims for greater ‘sensibility’ for a flourishing existence are necessary if we are to explore any educational implications in a productive way. Emotion is only one feature being raised by this faulty dualism, we need to explore what might constitute a more sensual and actively experiential form of life and expand on the idea of what it is for humans to flourish.

This piece therefore begins by reminding us of the central features of life that create our direct physical experiences of living, suggesting that we need to re-instate the body as intrinsic to mind and vice versa. This reinstatement is needed to understand how humans can fully flourish in the sense being advocated here. The deeply complex relationship between senses, feeling and understanding has been explored through a number of philosophical lines of thought but it is within pragmatist thinking that I locate my argument concerning this relationship. However, as Jim Garrison points out, “The ethos of pragmatism is extremely open, tolerant and accommodating; it evades attempts to totalize it into a single dogmatic vision” (Garrison and Neiman 2003: 21). For that reason, I need to highlight the particular dimension that has attracted my thinking for this piece. This rests on John Dewey’s idea that education should “release the human potential for growth” (Garrison and Neiman 2003: 28). To be able to grow, or to flourish, centres on living well with, in particular, the ability and desire to engage wholeheartedly with meaningful experiences that occur. Flourishing means being able to appreciate, desire and consciously pursue embodied activity that generates meaning. However, cultivating a richly meaningful life that entails the capacity to *grow* through encounters with the world could easily be seen as an individualistic, amoral aspiration for a singular, self-absorbed life, but pragmatic thinking, requires us to attend to the inevitability that humans exist as social beings. Thus, Garrison goes on to stress how, for Pragmatists, “Minds and selves emerge socially in critical and creative dialogue with the rest of the community” (Garrison and Neiman 2003: 22). And earlier, reminds us that: “The emphasis on consequences provides the starting point for almost any pragmatic analysis” (Garrison and Neiman 2003: 21). Particular qualities of living and thought are therefore significant in this conception of human flourishing but these rest on a rich and deep bed of human affiliation and practices, and thus echo the Aristotelian notion that social life in a community is a necessary condition for complete flourishing as a human being.

With Aristotle’s stress on human practices and the significance he gives ‘phronesis’ (Aristotle 2000) we should briefly consider the role practical reasoning might play in the ability to flourish and be able to work through consequences. For Amelie Rorty, it is important to remind ourselves that all conjecture in being able to see possibilities involves what she calls ‘constructive reasoning’. The ability to imagine things other than they are in our lives draws on imaginative strategies that help humans and communities progress by considering the consequences of hypothetical situations (Rorty 2009). Martha Nussbaum extends the civic significance of the imagination to being able to ‘sympathise’ with the other in what she calls the ‘narrative imagination’ (Nussbaum 2009). This means that flourishing, or the ability to ‘grow’, requires a range of abilities that allow for rich engagement in practices. Hence:

“We are all fully embodied ‘live creatures’ or ‘body-minds’ who interact with our environments in ways that involve intense phenomenological presence and sometimes also creative practical imagination” (Haskins 2019: 452).

A return to the significance of embodiment, sensuality and the imagination in human flourishing helps to turn the argument towards aspects of human practices with both wide ranging prevalence and also, high levels of accomplishment. As one of the leading pragmatists, Dewey’s philosophical writing and work on education arguably culminates with his later writings and conceptualisation for aesthetic experience. By exploring and amplifying aesthetics’ strong connection to embodiment, it will be possible to reassess a particular notion of performance (previously commonly associated with the neo-liberal stress on performativity) and thus advocate alternative views of how the quality of educational practice might be improved as we move into the future and emerge into a post-Covid 19 world.

## Life as Embodied and Experiential

To some degree an emphasis on senses and experience could merely be a description of the first phase of our presence in the world. From our visceral beginnings, experience and understanding of the world grows through what we see, smell, hear, taste and feel. Babies learn through their senses and the actions they are able to make within the bounds of their environment. The assumption is often made that with maturity and education, human capacity for thought and activity can develop but eclipsing some of these primal forms of interaction with the world becomes a necessity for that to happen. However, there is a well-established and alternative line of thought that prizes these early beginnings as the basis for an aesthetic dimension of living which is a fundamental human need throughout life.

“Nearly all the early shaping responses of human life are aesthetic in character, bringing through pleasure, pain or a diffuse sense of well-being, intimations of the nature of our common world. Long before we are rational beings, we are aesthetic beings; and we remain so, though often undeveloped and unsubtle, till ultimate insensibility defines the end of individual life. For death, in the precise words of Philip Larkin, administers ‘the anaesthetic from which none come round” (Abbs 1989: 4).

The sense of aesthetic introduced here by Abbs stands in contrast to the idea that aesthetic experience is linked solely or even primarily to the arts. Senses and feelings are seen as the common basis of all life’s experience in any situation. Their value and significance pervades our living throughout human existence, so that, as Mark Johnson explains, it is “via the aesthetics of our bodily senses, (that) the environment enters into the very shape of our thought, sculpting our most abstract reasoning out of our embodied interactions with the world” (Johnson 2007: 154).

If engagement with the world through the senses begins life’s journey, the value it might have for continued learning and wellbeing needs some exploration. Johnson goes on to argue that the value of embodied experience is linked inexorably to the fundamental human search for meaning, where meaning becomes possible through our emotions, movements, feelings and perceptions (Johnson 2007: ix). This can be seen when young children respond to the sensory stimulations afforded them in situations in which they find themselves. In general, they become active and experiment in ways that help them discover their own limitations. This kind of exploration allows patterns, familiarity and recognition to emerge. It is easy to observe how a baby’s focus seems to instinctively find interest in the human face in the weeks after birth. Carers around a newborn baby seem naturally drawn to respond and this leads to the baby mirroring expressions and even vocal sounds in just a very few months from birth. Before a baby can independently move her body across a room, these early beginnings of ‘mimesis’ (Scaramuzzo 2016) develop quickly into dexterity with gestures, grasping and giving of objects alongside developing proto language in mimicry of sounds. As young children use exploratory play to become familiar with the space, objects and people surrounding them, they begin to make sense of their world. John Dewey’s illustration of a child learning to avoid a flame through experiencing a burn, epitomizes how meanings can emerge through such situated experience. By seeing the flame and then reaching to touch it, the child learns what heat and pain mean and the object (the candle flame) has meaning for the child: light connects to heat, and in this instance, touch means pain (English and Doddington 2019: 415). In later life, imagine a hill walker as they encounter a storm. Whatever their understanding before, the sense of rocks, steepness, cold and exertion gain real, lived meaning for the walker as they struggle upwards. This direct experience may serve to enhance later activity as they draw upon this physicality or sensuality in imagination. Perhaps in appreciating or creating music, sound effects, in dance or while encountering visual depictions. These examples of ‘lived’ meaning point to the presence and immediacy of embodied aesthetic experience. They illustrate a fusion of actions, feeling and meaning. In turn they also have the capacity to feed and enhance the meaning of more abstract encounters such as when learning about the science of flammable material or the geographical study of a mountainous environment.

One significant feature of these illustrations of life experience and embodied learning or creation of meaning, is the sense in which the participant, to continue in Deweyan terms, is both ‘doing’ and ‘undergoing’ during the experience. Dewey is at pains to explain these strongly related dimensions of experience with care, in order to clarify how important and relevant they are to education. It is the embodied relationship and balance between doing and undergoing that creates meaning. However, the nature of how this duality stays in harmony is crucial. He warns that any imbalance can render the experience of little educational worth. It means, for example:

“Zeal for doing, lust for action, leaves many a person, especially in this hurried and impatient environment in which we live, with experience of an almost incredible paucity, all on the surface” (Dewey 1934/2005: 46). And in contrast:

“The sentimentalist and the day-dreamer’ may have an ‘excess of receptivity…., (But) the mere undergoing of this and that, irrespective of any meaning…..crowding together of as many impressions as possible (is no) more than a flitting and a sipping……(thus) nothing takes root in the mind when there is no balance between doing and receiving” (Dewey 1934/2005: 46–47). The degree to which aesthetic experience needs attentive ‘management’ is becoming clear.

While the stress given to educative experience as being embodied is perhaps relatively straightforward to warrant in childhood, a number of the writers who draw our attention to this dimension of existence argue that, as a feature of our aesthetic awareness, understanding the significance of seeing ourselves as embodied has value way beyond early childhood. From the handling of weights, shapes and blocks to develop basic mathematical concepts, to learning to play a musical instrument or enacting Shakespearian dialogue, embodiment is fundamental to practices involved in school learning and knowledge acquisition. The argument is that the bodily ways children find meaning are carried forward into adulthood, and actually “underlie and make possible, our mature acts of understanding, conceptualization and reasoning” (Johnson 2007: 33). Thus, to recall Abbs’ original claim, this suggests that a process which seeks to educate individuals towards mature acts of understanding, conceptualization and reasoning, rests and builds upon the fundamentally human dimension of our existence we have begun to identify as aesthetic experience.

## Aesthetic Experience and Education: How Can Somaesthetic Experience Educate?

It is important at this point to reiterate that the term ‘aesthetic’ here is understood as identifying any form of sense or soma-based experience which therefore includes, but also extends beyond, experience in the arts. For Dewey, aesthetic experience marked the very epitome of educative experiences for it has the power to “reveal human experience in its full development potential” (Fesmire 2019: xxx). Dewey explains that with senses fully engaged and an attitude that is open to the new or unfamiliar, balanced with a growing capacity for activity, imagination and reflection, an individual is primed for educative experience. Dewey develops the sense in which this constitutes *aesthetic* experience by identifying how the qualities of aesthetic experience include a vitality and immediacy as the individual struggles to find meaning and how this form of experience rounds itself towards a sense of completion or consummation (Doddington 2017). As implied earlier, these features of aesthetic experience, the undergoing and the doing, the feeling of being alive, and a sense of fulfilment can be echoed in problem solving and forms of enquiry as well as in artistic engagements. In teaching history or science, rather than delivery of propositions or facts, the details and meaning of a given event or scientific theory can be brought alive or conveyed through practical and embodied experience by devising group investigations or by using role play, for example. But the sense in which we can accept these kinds of learning practices as *education* depends on understanding Dewey’s related notion of growth. Dewey argues that a situation that calls on previous experience and encourages the learner to actively search for, or construct meaning for themselves, creates a double benefit. “In the aesthetic experience so defined by Dewey there is for the reader, maker or viewer an active process of clarification, even purification, as meanings and prior experiences come together in the coherence of present perception” (Grierson 2017: 1256). And an embodied understanding also transforms the individual because it extends into future experiences by creating an appetite and capacity to further one’s learning through full-bodied engagement. “Aesthetic experience epitomises our meaningful bodily transactions with our environments” (Haskins 2019: 454). For Dewey, aesthetic experience so defined, lies at the heart of educational practice.

Richard Shusterman has developed Dewey’s pragmatic aesthetics concerning an emphasis on the senses and in particular on embodiment, in his work on *somaesthetics*. Shusterman observes that modern life can easily supress the human ability to experience the world aesthetically. He explains that bodily perceptions and habits, like our use of language and behaviours, can become entrenched and sink from explicit awareness, forming a diminished background for life. Shusterman argues that physical exercise, meditative awareness and practices such as yoga or the Alexander Method offer the opportunity to improve our bodily movements and feeling and strengthen the basic foundation from which we think and act by enhancing somatic self-knowledge and encouraging greater self-care. The key lies in our ability to see the importance of increased aesthetic awareness, for “Bringing aesthetics closer to the realm of life and practice…..entails bringing the body more centrally into aesthetic focus, as all life and practice—all perception, cognition, and action—is crucially performed through the body” (Shusterman 2012: 140). With our aesthetic focus on the body heightened, our engagement with any situation or environment in which we are placed is enhanced and the sense in which we can wholeheartedly engage is increased. This suggests that the sense in which we gain meaning from and feel enlivened by experience is also developed and so, our ability to grow and flourish may be strengthened.

Embodiment is central to a full sense of awareness but is also central to our capacity for gaining deeper understanding. Looking at the philosophy of language, Mark Johnson’s work offers a detailed critique of a prevalent representational approach and introduces the idea that it is metaphor that underpins much of our thought, including abstract thought. He looks at basic bodily metaphors that give language meaning, showing how commonly we speak metaphorically. He exemplifies this by reminding us how we might describe ‘difficulties’ as ‘burdens’, understand ‘affection’ in terms of ‘warmth’, or ‘understanding’ in terms of ‘seeing’. Extending the everyday use of metaphor, Johnson illustrates the experiential basis of theoretical understanding in areas such as arithmetic (as object collection), or music (as a ‘moving force’). His claim is that abstract concepts are defined primarily by conceptual metaphors. For Johnson, metaphors therefore lie “at the heart of human understanding and reasoning” (Johnson 2007: 200), arguing that they “make it possible for the theories to make sense of our experience” (Johnson 2007: 204). He also reminds us that we must look beyond the spoken and written word here, for “the idea that only words can have meanings ignores the vast stretches on the landscape of human meaning-making” and “denies the status of *meaning* to most of the meaning-making that occurs beneath our conscious awareness and beneath representational structures” (Johnson 2007: 207). Johnson does not explicitly develop his thesis for education in this book, but the implications are clear. If understanding the abstract material that features within a curriculum is seen as important, then supporting students to find that material meaningful is a crucial dimension of educational practice. While Shusterman’s work sees the relevance of somaesthetics to education resting in activities which increase student’s conscious awareness of their bodies (2004), Johnson’s focus is on the meaning of the body as integral to what he terms ‘the aesthetics of understanding’ (2007).

As opposed to an exclusive emphasis on developing the mind, it is possible to argue that education in schools should counter that view as a distortion and offer instead, opportunities for cultivating the aesthetic nature of being. The trend towards education in the open air via forest schools or in adventure education is frequently justified by reference to risk and physical wellbeing but using a somaesthetic lens reveals the aesthetic dimension more clearly. Time spent actively in nature for example, can present aesthetic challenge and encourage “flexible, sensitised habit to generate growth and desire for (further aesthetic) experience” (Doddington 2019: 130). And while it is clear that aesthetic sensibility extends beyond artistic practices, the arts clearly provide the opportunity for embodied understanding of aesthetic experience and its value. The arts distinctively call for a duality of sensual participation and spectatorship, to being both openly receptive to the new as well as active in how one takes part. We participate even as static ‘audience’ to conjure meaning from what we see and hear. The arts therefore lend themselves to being taught in ways that encourage sensual and embodied engagement. In the most obvious of examples, literature and poetry brought to life by drama activity or image-making give opportunities for revitalising the critical interpretation of texts to enhance understanding and meaning. Opportunity for shared study and embodied exploration in this way can create a different kind of environment where “the sharing and expression in different forms of what is authentically felt or perceived would be valid and encouraged and the ideas of standardization, levels and measurements would fade with the educator’s interest focused on enabling the young to extend their capacities to see, feel, hear, move and bodily enact their ideas” (Doddington 2015: 64).

While the idea of attaching value to the development of aesthetic sensibility is not new, there are European examples of projects where this has been an explicit aspiration (Urpí and Doddington 2015). Yet other educationalists have developed practices of highlighting the value of embodied aesthetic experience as a way to encourage imagination and a sense of belonging. As an example of real practitioners striving to conceive of education beyond the neo-liberal vision, an organisation based in Cambridge called ‘Cambridge Curiosity and Imagination’, (CCI) (https://www.cambridgecandi.org.uk/) has done extensive work with schools over the last fifteen years. Formed in the face of what was seen as ‘continual erosion of freedom for children’ and ‘a commitment to the transformative impact of the arts’, the organisation works with artists and schools, largely in outside spaces. In a recent project entitled ‘Artscapers’, artists and movement specialists worked with children and their communities to explore the development of a new mixed urban and academic community on University land, known as the North West Cambridge Development, now named Eddington. Children traversed and experienced the ecology and development of the site, made designs of sculptures and art works and were given time and generous materials to work at real problems with parents, teachers and artists who encouraged and valued the children’s ideas. The experience is recorded and celebrated in ‘Artscapers: Being and Becoming Creative’ (Ayliffe et al. 2020) and the manifesto, constructed at the close of the project by all who took part, including the children, was shared in the UK parliament with the All Party Parliamentary Group for Art, Craft and Design in Education. During the recent Covid pandemic, this group has continued to support families and those schooling Key Worker’s children with craft/creativity packs and imaginative use of natural, outside locations. The take-up of this support has been widespread. This, alongside the extensive media promotion of physical and creative activity, crafts, gardening, and closeness to nature, seems to highlight the real human need for embodied activities and aesthetic experience during these recent, difficult and ultimately, tragic times of the Covid 19 pandemic.

In other empirical work designed to verify the value of aesthetic experience and to explore ways in which teachers might provide the opportunity for students to have such experiences, P. B. Uhrmacher uses qualitative research to describe six themes arising from Dewey’s account of aesthetic experience. “*Active engagement, sensory experience, connections, imagination, perceptivity,* and *risk taking* are conditions that encourage aesthetic experiences” (Uhrmacher 2009: 635). Creating tasks which require active participation and call upon the senses can encourage strong engagement. But these are insufficient on their own if those tasks are designed to allow for connection to other understandings or previous experiences. If the hope is for students to make sense and create meaning in the new material, then students need to be encouraged to imagine what might be possible and search for meaning by being perceptive. To realise there may not be a ‘right’ answer, to think, be self-critical and question one’s own assumptions takes confidence and courage—hence the classroom environment has to be both stimulating and secure, encouraging students to be bold enough to be open, *and* to take risks. With these aspects of aesthetic experience in mind, Uhrmacher’s research found that “The more a teacher is able to encourage a classroom community that embraces these themes, the more likely it will be that the students, and the teacher, grow from the experience” (Uhrmacher 2009: 635). By way of attempting to further justify the idea of aesthetic experience, the writers go on to suggest: “it is these experiences that seem to support deep engagement as well as memory retention, an increase in knowledge, student satisfaction, meaning making and creativity” (Uhrmacher 2009: 635). However, with this list, the research seems to slide into bolstering the value of aesthetic experience by resorting to more hackneyed and familiar warrants for particular forms of pedagogy or curriculum content. I believe this takes us away from the more subtle understandings of Dewey’s notion of aesthetic experience and education whereby knowledge acquisition, retention and satisfaction may be incidental but are not forwarded as the main criteria for successful educational experience.

If the argument for features of aesthetic experience being vital to understanding has some validity, the sense in which pedagogy should take account of it needs more clarity and definition. But before this can be further explored, one dimension that has not been explicitly analysed, needs attention as a fundamental justification for basing education on aesthetic experience. So far, the discussion has appeared to focus on the importance of aesthetic experience for individual growth. And it is widely argued that enhanced aesthetic experience for learning and in life generally, can have real personal value. But Shusterman briefly makes the point that not all somaesthetic experience, for example, is necessarily undertaken in a solo situation and when it is experienced as part of a group, there are further benefits to note. “Communal energy greatly enhances not only the individual’s meditative power but also social solidarity” (Shusterman 2012:15). So the idea that aesthetic experience may have a place in fostering and strengthening solidarity warrants further consideration.

## School as a Community for Aesthetic Experience and the Role of the Teacher

The argument in this paper so far has sought to establish the sense in which the senses and embodiment is the grounding for human flourishing, noting how this has been previously neglected in discussions concerning education. As we have seen, more recently there has been a growing acknowledgement by some philosophers, of the sense in which humanity relies for *meaningfulness* on embodied, aesthetic experience (Shusterman 2012; Johnson 2007). And as we transpose these ideas into the practices of education, we cannot fail to acknowledge that much of the activity designed to be educational in school takes place in the context of others being present, we might say, is experienced communally. A call for aesthetic sensibility to be at the heart of educational practice means that we need a clear understanding of what constitutes this form of sensibility, which I have tried to set out in the previous sections. However, we also need to understand what it might mean for this understanding to help form a school community and pervade the thinking that underpins that community.

Any community is usually bound together by a series of common practices and beliefs. In the case of schools, Dewey explains this will include: “aims, beliefs, aspirations, knowledge—a common understanding—like-mindedness”. However, fundamental to this is “The communication which insures participation in a common understanding …..which secures similar emotional and intellectual dispositions – like ways of responding to expectations and requirements” (Dewey 1916/68: 4–6). This is not to say that ‘like-mindedness’ will exclude those with different views, for openness and dialogue is vital to sustain any coherent and living community and as Jim Garrison explains, while every individual has unique potential for Dewey, “Individuals need otherness and difference to develop their distinctive potentials” (Garrison 2019: 363). So ‘like-mindedness’ simply means that, in the case of a school community, what is considered to constitute education may be very broad and diverse but needs to be collectively compatible for there to be a coherent sense in which the purpose of the community is a *shared* understanding. Compatible understandings and beliefs in the value of experience that is aesthetic is likely to be necessary to strongly support some of the features of pedagogy discussed so far. For aesthetic experience to be encouraged, developed and deeply inherent in the learning community of a school or a classroom, it needs to be both collectively embraced and to become pervasive as an explicitly shared value. We need therefore a little more detailed consideration to unpack how this might emerge.

Pedagogy which focuses on creating experience with a balance between open receptivity and participation is frequently characterised as teachers creating environments where learners can be ‘active’ in their learning. Our earlier discussion of the aesthetic suggests that both teachers and learners need to embody a particular kind of openness to impressions in order to allow for experience to be fully *felt*. However, the aesthetic also alerts us to the richness of being active in ways which call upon a full range of expressive powers to find “meaning that (might) exceed what can be expressed through language” (Fesmire, 2019: 208). When Dewey develops his idea of the importance of associated living, and thus his belief in democracy, he stresses the need for good communication within a community and argues that “The very process of living together educates” (Dewey 1916/68: 6). The tendency here for some educationalists, is to focus on the verbal dimensions of communication which thereby justifies the significance of, for example, classroom dialogue. Although verbal language is extensive in education and obviously a vital facet of communication, Dewey also reminds us that ‘communication is like art’ and “is the foundation and source of all activities and relations that are distinctive of internal union of human beings with one another” (Dewey 1934: 348). He goes on to explain that the collective nature of a wide conception of communication beyond mere language is important for the way it “enlarges and enlightens experience; it simulates and enriches imagination; it creates responsibility for accuracy and vividness of statement and thought” (Dewey 1916/68: 4–6).

An enlarged conception of communication can help us see the extent of the value of some simple classroom practices. A teacher presenting an image to a group or class will want them to find meaning in the image. Simply asking what they see is one way of exploring the image but in addition, asking the group to discuss what questions they would like to ask of the maker or those present in the image, may be ‘in role’, arguably asks the group to look a little harder and think aloud and together. Similarly asking a group to construct an image that encapsulates the essential meanings or significance of an event, in history for example, calls upon the group to use their bodily aesthetic sensibilities. Image making can be used in schools to ascertain how meaningful students find the event and therefore can allow teachers to gauge the extent of their understanding. Placing these kinds of activities in a group rather than as an individual exercise points out the sense in which collective activities can have value. Participants often remark how fitness and yoga classes give a different experience to the same activity undertaken as a solo activity. Some point to stronger motivation and a greater sense of inclusion and belonging as being among the benefits. Similarly, group work, games and physical exercise in schools have the potential to generate a collective sense of sociability and vitality. If we add to this the use of imagination and the generation and sharing of thoughts and ideas in a successful group-based presentation, creative dance or drama session, the potential for encouraging aesthetic experience and full-hearted engagement seems to increase.

Schools undeniably centre around many different kinds of collective experience and would seem to have the potential for cultivating a rich sense of communication necessary for community. But the emphasis on generating collective vitality or ‘communal energy’ raises an interesting and perhaps rather neglected feature of classroom experience. This is not to say that the day-to-day practice within classrooms can, or should be, a continuous, emotionally heightened experience. But if we return to the characteristics of an aesthetic experience and its connection to flourishing, we are looking for experiences that can round to a felt consummation, that thus offer meaning and give a sense of coherence to a learning experience. Dewey argues that observation of children suggests that they are not necessarily, or naturally, inclined to consummate or complete experience in this way. However, he comments they *are* able to enter “into an activity that calls the whole self into play” (Dewey 1934: 61). With an emphasis on seeing how this might be a *shared* experience, we are looking at how the beginning of some teaching or learning episode might be “initiated by a tensive excitement that compels us to focus on the unfolding experience” (Alexander 1998: 14). This is what Dewey means when he argues that an experience that is aesthetic, can ‘seize’ our attention. This has implications for the teacher planning learning experiences that are designed to enthral or intrigue, exhilarate or challenge, in ways that give the experience impulse. But then there are also implications for how the teacher can help ‘orchestrate’ or give shape to the experience of her planned activities and in particular help to draw experiences round with a sense of completion. Like all artistic creations involving the active participation of others, none of this can be fully pre-determined. It therefore calls upon the teacher to be receptive, observant and present, to listen carefully as she ‘explores’ the material alongside her students and to be responsive and conscious of ways in which the experiences may be deepened, extended and resolved with a sense of fulfilment at completion.

For Dewey, an educative experience should have the strength to not only call upon previous experience to help make sense of what is encountered, but also create the appetite and continuity for further educational experience. Dewey is clear that this development is not about creating ‘just more energy’. Educative experience evolves through a “transformation of energy into thoughtful action”. This means that “the junction of the new and old is not a mere composition of forces but is a re-creation in which the present impulsion gets form and solidity while the old, the ‘stored’ material is literally revived, given new life and soul through having to meet a new situation” (Dewey 1934: 61). Personal and individual ‘stored’ understandings can be shown, shared and pooled with group activities to aid the discovery of meaning. And similarly, the translating of a stimulated experience into communicable forms of expression, analysis or creation, can be shared. Time is also a consideration here. Teaching and learning episodes that are initiated can span moments or days before they arrive at a desired-for fulfilment or completion. Stephen Fesmire, writing of general, cultural and situated experience explains: “as with the act of writing an essay or the event of childbirth, so situations are temporally spread out. From composing a musical score to negotiating a peace treaty, the situational ‘spread’ includes the immediately felt echo of the prior flow of experience and the dawning sense of the future flow” (Fesmire 2019: 423). Calls for ‘pace’ and bite-sized single, objective-led lessons would seem to mitigate against this possibility of taking time to allow for experience and understanding to culminate.

By drawing together some of the points made so far, it is possible to imagine the kinds of school experiences that could exhibit features of aesthetic experience and highlight a sense of communal energy. The embodied nature of collective singing when it is joyous or emotionally powerful, the struggle to trek and explore the wildness of outdoor space together, the demonstrated science experiment or the creation of drama for performance all have obvious potential here. But there can be quieter moments: in attending together to a class member’s artwork or listening to a student’s account of something they have a passion in, as in the classic episode recounted in Barry Hines’ novel “A Kestrel for a Knave” (1968) or Ken Loach’s film of the book “Kes” (1970), when a quiet Billy Casper comes to life describing how he finds and trains two kestrels. Yet the range of possibilities, from ‘give an account of your interest’ to singing together, can also be introduced and encountered as dreary, pedestrian activities, becoming routine, too rushed or banal and uninspiring. For this reason, as suggested earlier, it is the teacher who is crucial to orchestrating the qualities and complex dynamics of aesthetic experience.

It has been suggested that one way of capturing something of the complexity of this envisaged role for teaching is for it to be understood more as curating (Ruitenberg 2015). Ruitenberg explains the mutual features of teaching and art curating in terms of the *criticality* required of both activities and some of the mutual abilities and understandings they have in common. The features that may be relevant include the capability to have deep knowledge and commitment to a subject or specific material, together with powers to interpret, select and present that material in ways that are accessible. Ruitenberg suggests this can be seen as building a bridge between those encountering the material and the material itself. With her stress on criticality, she is encouraging teachers to acknowledge the power and authority they have to arrange and ‘style’ material that may be part of a curriculum syllabus that appears fixed but which may benefit from creative design to be more effective. One interesting dimension that she highlights is also the need for both curators and teachers to actually ‘create’ the publics that encounter works or material. She writes of both roles needing to enable ‘conversations and relations’ i.e. “the ability to bring people, experiences, ideas and works together in thought-provoking ways” (Ruitenberg 2015: 232). While there is much in this idea that seems to capture the role of the teacher, I believe there are some dimensions that may be missed in relation to creating the aesthetic nature of educative experience as I have suggested earlier.

Ways of communicating within a community that is rich in aesthetic experience opens up the possibility of transforming and enlarging students’ perspectives. As with viewers in a gallery for example, there will be difference in response but also recognition and similarities with their understanding linked to the material being viewed. In a *shared* experience, students or viewers may encounter resistance to their thoughts as well as discover new ideas. They might create meaning in a merging of views, or individually experience minor shifts, perhaps in beliefs, values and thinking. But it is clear that if education were to embrace the significance of aesthetic experience, the responsibility for teachers to engage and manage the environment, the relationships and the content of the curriculum is great and may go beyond the idea of curating. Another suggestion is that one might view the role of the teacher more as if they were an artist (Garrison 2010), (English and Doddington 2019: 427). As against programmed teaching to pre-ordained achievement targets that are so widespread throughout education in the form of specific learning objectives, this kind of teacher needs perception and insightful knowledge of the present context. They also have to imagine likely consequences and look ahead. Not only do they need to understand what a group of students already knows and can do, they need to understand the historical, social and cultural identity that each child brings to the learning situation as well as “knowledge of what is needed for (each) child to become part of, and contribute to, the growing community of the classroom” (English and Doddington 2019: 427). The teacher may initially work on the material of her teaching, as a curator might consider the material they have to play with, planning how it can become a vehicle of understanding for her students/viewers. But if teaching is to focus on aesthetic experience for meaning, teaching needs not only to be thoughtfully planned but also practiced as *improvisation*, working with everything that “emerges serendipitously in the process of creation – the insights, the shifts of attention, the fortuitous associations, the unforeseen opportunities and promises instantly perceived” (Jackson 2000: 187–188 in English and Doddington 2019: 428). In more detail, Jim Garrison suggests something of the aesthetic nature, mutuality and unpredictability of educational practice with what he calls ‘the teachable moment’ He writes:

“I propose understanding teachable moments as the times when spaces open for the student and teacher to interact in a synchronistic and dynamic rhythm. It is like having a good dance partner. During these moments there is a special or enhanced quality of intimacy, openness, and creativity” (Garrison 2010: 122).

This idea of seeing both the teacher and the student as intimately linked to this degree in the encounter with material, seems to take us away from the notion of the teacher as curator. The nearest analogy arising from the points I am making is perhaps more like the engagement between a vibrant company of actors working with a skilled and democratic theatre director. Here, while the director can take overall responsibility for the play, or ‘interplay’ that emerges in rehearsal, they can also be open and responsive, encouraging genuine participation and contribution from the ‘ensemble’ of actors. Meanings are not set firmly within a text or script and can emerge through tentative experiments and improvisational approaches in the theatre. Furthermore, meaning and significance can be suggested but cannot be projected directly to be received, intact, by an audience. Meanings will continue to emerge in rehearsal and again in the live enactment for those participating in the encounter as audience and even as actors.

When a teacher interacts with a student or students, or a student interacts with another student or students, the communication can be subtly, or explicitly, public. In groups, interactions are therefore invariably *performed* in this sense, before others. This kind of performance is meant in contrast to the underlying thrust of measurement and performativity cited at the beginning of this article. It is also necessary to distinguish it from the idea of performance pedagogy which is more centred on the idea of a teacher performing as an actor or functioning more as a projectionist. I am proposing therefore, a very different and alternative sense of performance that is more like a safe ‘sharing’ that offers the opportunity of communion. Covid has left ‘lockdowned’ individuals and communities craving for socialisation, fresh sensory, aesthetic experience and closeness with others. It is in risking the performance of our ideas and ourselves in communion with ‘like-minded’ others, that gives the possibility of growth. But this is not to advocate just a bland and benign acceptance of everything produced, for as we noted earlier, developing one’s potential and growth needs wholehearted and responsive encounters with otherness and difference. But just as there is hope for a less frantic return to living with greater mutual respect and acceptance of what is truly valuable to human life after Covid, maybe education too has possibilities, whereby schools could embrace the value of aesthetic experience in the safety of the ‘created’ public domain that constitutes school life.

Performing *within* one’s community, whether that is the group, classroom, the school or the locality, can be a safe, shared, collective experience. And with warnings of the apparent extensive cost to the mental health of both children and adults, a longing to feel safe has never been more apparent than it is now, post-Covid. With a shared atmosphere of commitment to aesthetic experience, forms of expression might emerge that strike “below the barriers that separate human beings from one another” (Dewey 1934: 282). Where an ‘audience’ is fully engaged and collectively value the nature of the activity, there is the potential for all to share in the vitality and appreciation of the wide range of embodied and sense-enhanced experience that can manifest itself in cohesive groups. We know that interactions and relationships that are responsive and receptive can contribute strongly to the collective nature and communal ethos of a school or classroom and encourage a sense of solidarity. The argument here is that the aesthetic nature of living described from birth can continue throughout life and can be the source of expanding our horizons, understanding and sheer pleasure in being alive. The final suggestion made is that, like the significant role mimesis plays, first in early communication and then throughout later life, aesthetic experience as ‘shared’ experience, can be seen to play a vital role in developing the strengths and highly necessary abilities needed for humans to bond, relate and co-exist (Scaramuzzo 2016). If solidarity is a valuable feature of community, I have tried to show that it may be worthwhile to re-configure pedagogy so that aesthetic experience is seen as central to educational practice and that teachers have a crucial ‘directing’ role here. Just as Nussbaum claims that imaginative reasoning is capable of being developed through the study of the arts and literature, my argument has been that, student’s sensory and bodily enactments with the world, have the potential to be humanely educated in ways that both promote human flourishing, *and* foster better forms of cohesive and enriched social living. And, while the first is important, this last claim is perhaps one of the most pressing needs for our present world and the world we might wish to live in, in the future.
